# Predicting school readiness program implementation in community-based childcare centers

**DOI:** 10.3389/fpsyg.2022.1023505

**Published:** 2022-12-15

**Authors:** Leah J. Hunter, Benjamin L. Bayly, Karen L. Bierman, Janet A. Welsh, Julia M. Gest

**Affiliations:** ^1^Department of Psychology, University of Pittsburgh, Pittsburgh, PA, United States; ^2^Department of Agricultural Economics, Sociology, and Education, College of Agricultural Sciences, The Pennsylvania State University, University Park, PA, United States; ^3^Department of Psychology, The Pennsylvania State University, University Park, PA, United States; ^4^Edna Bennett Pierce Prevention Research Center, College of Health and Human Development, The Pennsylvania State University, University Park, PA, United States

**Keywords:** school readiness, implementation, childcare center, social–emotional learning, literacy skills, preschool, teacher coaching

## Abstract

**Introduction:**

Targeted curricular interventions can increase preschool program quality and boost children’s academic and social–emotional readiness skills, but variable funding and weak organizational infrastructure in many community-based childcare centers may reduce the effective implementation of these programs.

**Method:**

This study examined individual teacher and workplace predictors of the REDI program implementation, a targeted school readiness program that was adapted to support delivery in childcare centers. REDI was delivered by 63 teachers in 37 community-based childcare centers with center directors serving as local implementation coaches.

**Results:**

Results showed that individual teacher factors (e.g., teaching skills and receptivity to intervention consultation) predicted the quality with which REDI activities and teaching strategies were delivered, and workplace factors were important predictors across multiple implementation indicators.

**Discussion:**

Practice and policy implications for improving intervention implementation and corresponding program quality in childcare centers are highlighted.

## Introduction

High-quality early childhood education (ECE) promotes school readiness skills and fosters long-term school success, with heightened benefits for preschool children from low-income families (Yoshikawa et al., 2013; Phillips D. A. et al., 2017). Access to preschool programs has increased over the past two decades, as has the inclusion of social–emotional learning elements in early learning programs (Bryant et al., 2021), but wide variations in program quality remain a significant concern (Ackerman and Sansanelli, 2010; Donoghue, 2017; Pianta and Hamre, 2020). Community-based childcare centers are especially variable, with average teaching quality levels significantly lower than those in publicly managed programs such as Head Start and school district prekindergarten programs (Burchinal et al., 2008; Dowsett et al., 2008; Hillemeier et al., 2013; Bassok et al., 2016a).

Research conducted during the past two decades suggests that preschool program quality can be enhanced by enriching classrooms with evidence-based curricula and providing teachers with corresponding professional development support and coaching (Yoshikawa et al., 2013; Phillips D. A. et al., 2017). However, this research has focused almost exclusively on publicly-funded Head Start and public prekindergarten programs (McCormick et al., 2015; pre-k; Phillips D. A. et al., 2017). Childcare centers have more variable organization and funding structures than publicly-funded programs, with fewer resources and regulatory supports, which may reduce their capacity to adopt new evidence-based programming (Ackerman and Sansanelli, 2010; Bassok et al., 2016a; Whitebook et al., 2018; McCormick et al., 2022). Indeed, initial efforts to “scale up” evidence-based preschool programs more broadly in community-based childcare settings have encountered significant implementation challenges (Baker et al., 2010; Yurdon et al., 2016). Research is needed to better understand the factors that facilitate or impede the effective implementation of evidence-based programming in childcare contexts to ensure these programs can be brought to scale successfully. The current mixed methods study addressed this issue by exploring teacher and workplace factors associated with the quality of implementation of an evidence-based school readiness program (the Research-based Developmentally Informed [REDI] program) in childcare classrooms.

## The need to improve preschool programming in childcare centers

Childcare centers serve one-third of children attending preschool in the United States (NCES, 2020). Unlike Head Start or school district pre-K programs, childcare centers represent separate, diverse entities operating within a de-centralized system that lacks common standards for accreditation or operation (Ackerman and Sansanelli, 2010; Bassok et al., 2016a). Childcare centers operate under varied management structures, ranging from for-profit corporations and non-profit cooperatives to small, independently-owned and operated businesses (Ackerman et al., 2009). They are often under-resourced, with average teacher salaries and benefits well below those provided in publicly-funded programs (Whitebook et al., 2018; Johnson et al., 2019). Correspondingly, childcare teachers serving preschool children often have lower levels of formal education and training than teachers in publicly-funded programs, and typically experience higher levels of stress and job dissatisfaction (Bassok et al., 2016a; Whitebook et al., 2018). They leave their jobs at high rates and move to more well-funded positions when they can (Zaslow et al., 2010).

Not surprisingly, when compared on similar measures of observed preschool teaching quality, childcare centers show mean levels of emotional support and cognitive stimulation that are significantly lower than those documented in Head Start or school district pre-K classrooms (Dowsett et al., 2008; Hillemeier et al., 2013; Bassok et al., 2016b; McCormick et al., 2022). Evidence-based strategies that have proven effective at improving quality in publicly-funded preschool settings may also enhance the quality of childcare centers; however, these strategies are rarely studied in childcare contexts (McCormick et al., 2015; Phillips D. A. et al., 2017). leaving unanswered questions about the ways in which childcare teacher or workplace factors might affect implementation quality of the strategies.

## Implementing evidence-based strategies that boost the school readiness of preschoolers

Current research suggests that the most effective strategies for improving preschool program quality and boosting child school readiness outcomes utilize two approaches (Yoshikawa et al., 2013; Phillips D. A. et al., 2017). First, effective intervention approaches provide teachers with professional development support and coaching in high-quality teaching practices designed to boost emotional support, enriched language use, and instructional quality (Hamre et al., 2012; Pianta et al., 2020). Second, some effective approaches also increase child learning opportunities in the classroom by enriching daily programming with manualized, skill-specific curriculum components that provide lesson plans and sequenced learning activities (Jenkins and Duncan, 2017; Nguyen et al., 2018). These curriculum components are typically domain-specific (e.g., focused on early literacy, mathematics, or social–emotional skills) and are especially effective for boosting child skills in the targeted domains relative to more global curricular approaches (Jenkins and Duncan, 2017). The ultimate goal of these two approaches is to elevate levels of social–emotional support and cognitive stimulation in the classroom, and thereby accelerate the pace of growth in school readiness skills (Jones and Bouffard, 2012; Maier et al., 2022).

Key markers of implementation quality for interventions that use both approaches include: (1) completing the sequenced lesson plans as written, reflecting *adherence* to intervention guidelines, and (2) using the prescribed teaching strategies while delivering lessons and interacting with children in the classroom, reflecting *quality* in program delivery and generalized use of the recommended teaching strategies (Gearing et al., 2011). A limited research base suggests that the predictors of implementation quality may vary depending upon the facet studied (e.g., curriculum delivery adherence or teaching strategy quality) as described in the next section.

## Predictors of implementation quality in evidence-based preschool intervention

Domitrovich et al. (2008) proposed a multilevel framework to describe the determinants of school-based program implementation. Determinants included individual-level characteristics of the teachers who implement the intervention (such as teacher training and experience) and also workplace factors (such as school climate and administrative leadership) that provide a support system for the intervention. In the following sections, we review evidence regarding the association of teacher characteristics and workplace factors with adherence and quality of school readiness intervention implementation in preschool classrooms.^1^

### Teacher characteristics

#### Professional background.

Teacher education has been fairly well-studied as a predictor of preschool program implementation quality. Two studies have linked teacher education to intervention adherence. Teachers with an early childhood education background conducted more Banking Time dyadic intervention sessions to target children’s disruptive behavior compared to teachers without an early childhood specialization (Williford et al., 2015). The authors speculated that having a degree focused on early childhood increased uptake of the teacher-child relationship-focused intervention. In the second study, teachers with master’s degrees used the BEST in CLASS behavior management strategies more often than teachers with high school or associate degrees (Sutherland et al., 2018). However, only the BEST in CLASS (and not the Banking Time) intervention documented links between teacher education and the quality with which the intervention was delivered, possibly due to the demands of the 2-tiered intervention (Sutherland et al., 2018). Teacher education was not consistently related to implementation adherence or quality in multiple interventions that included classroom curricular lessons and strategies, including the Bloom Language Curriculum (Phillips B. M. et al., 2017), Building Bridges (Baker et al., 2010), Second Step (Wenz-Gross and Upshur, 2012), and the Head Start REDI (*Re*search-based, *D*evelopmentally *I*nformed) program delivered in Head Start centers (Domitrovich et al., 2009). These findings suggest that teacher education levels are generally not predictive of implementation for interventions that include guided classroom curricula, but they may affect uptake of new teaching strategies in more intensive intervention programs that focus on student–teacher interaction quality.

#### Teaching skills.

From a conceptual standpoint, foundational teaching skills, such as positive classroom management skills and proficiency in instructional support may foster high-quality preschool program implementation by reducing child disruptiveness and increasing student engagement. Further, teaching skills may accelerate a teacher’s capacity to adopt new teaching strategies by allowing teachers to build upon their higher baseline levels of competence and confidence (Gage et al., 2015). Supporting this hypothesis, pre-intervention observations of teacher-student interaction quality significantly predicted the quality of delivery of the preschool Second Step curriculum (Wenz-Gross and Upshur, 2012), the BEST in CLASS intervention (Sutherland et al., 2018), the Bloom Language Curriculum (Phillips B. M. et al., 2017), and the Getting Ready for School program (Marti et al., 2018). Pre-intervention teacher-student interaction quality also predicted adherence (number of lessons taught) in the Second Step curriculum study (Wenz-Gross and Upshur, 2012), but was not related to adherence in the other studies.

#### Responsiveness to intervention

Researchers have suggested that teachers put more effort into delivering an intervention when they feel comfortable with the intervention approach and are open to consultation and feedback about their implementation quality (Domitrovich et al., 2008). Consistent with this expectation, positive attitudes toward the intervention (measured *via* pre-intervention teacher self-report) predicted the quality of teacher delivery of a language-literacy skills intervention (Zucker et al., 2013) and the Bloom Language Curriculum (Phillips B. M. et al., 2017). Similarly, both Domitrovich et al. (2009) and LoCasale-Crouch et al. (2016) found that teachers who were more responsive to and enthusiastic about the coaching they received showed higher levels of quality when using the teaching strategies that were a focus of the intervention. Teacher receptivity to the intervention also predicted adherence in delivery of the Bloom Language Curriculum (Phillips B. M. et al., 2017). Conversely, teacher concerns about the intervention predicted lower adherence in delivering the Building Bridges curriculum activities (Baker et al., 2010).

In summary, prior studies generally suggest little impact of teacher education on implementation adherence or quality, more consistent support for baseline teaching skills as a facilitator of implementation quality (and sometimes adherence), and consistent associations between teacher receptivity toward the intervention and both implementation quality and adherence. With few exceptions, the studies cited examined intervention implementation in Head Start or public pre-kindergarten contexts, leaving unknown questions about the value of these teacher characteristics as predictors of implementation in childcare settings.

### Workplace factors

In contrast to teacher characteristics, workplace factors are rarely studied as predictors of preschool program implementation, but they may be key to understanding challenges associated with diffusing evidence-based programs in under-resourced childcare centers characterized by variable and generally low levels of infrastructure support. In the conceptual framework outlined by Domitrovich et al. (2008), school-level factors may influence intervention implementation either directly by the degree to which the intervention is supported at the administrative level, or indirectly, though the impact of the workplace on teacher morale. Several features in this domain distinguish childcare centers from publicly-supported preschools: classroom resources, teacher job satisfaction, organizational learning support, and workplace challenges (Dennis and O’Connor, 2013).

#### Classroom resources

The early learning standards of the National Association for the Education of Young Children (2002) specify the importance of adequate classroom resources to support the implementation of high-quality early education practices. Child-care centers vary considerably in their access to these resources due to the limited and fragmented funding streams they rely on (Ma et al., 2021). We found only one prior study that examined classroom resources as a predictor of evidence-based program implementation. Wenz-Gross and Upshur (2012) assessed the classroom environment with the Early Childhood Environment Rating Scale–Revised. This composite rating reflected the classroom space and furnishings, books and communication supports, activity centers and materials, and program schedule. It supported teacher adherence to the delivery of the Second Step program but was unrelated to implementation quality (Wenz-Gross and Upshur, 2012). The authors speculated that being in a more well-resourced classroom reduced obstacles to intervention delivery and boosted teacher feelings of efficacy and motivation to invest in improved programming.

#### Job satisfaction

Prekindergarten teaching positions pay less, offer fewer benefits (including less time off), and provide teachers with fewer opportunities for professional development opportunities than similar positions in public schools (Whitebook et al., 2018; Johnson et al., 2019). Teachers in these settings view their jobs as lower status jobs (Morrissey et al., 2007) and often express higher levels of stress and job dissatisfaction than their counterparts working in public schools (Bassok et al., 2016a; Whitebook et al., 2018). Prior research suggests that when teachers feel more supported, satisfied, and effective at their jobs, they implement a new program more effectively, whereas job-related stress and burnout are associated with reduced implementation adherence and quality (Ransford et al., 2009; Baker et al., 2010).

#### Organizational learning

Research suggests that a key characteristic of high-quality ECE programs is a high level of support for staff professional development and program improvement efforts (Ehrlich et al., 2016). Referred to as organizational learning (Bryk et al., 1999), this construct reflects the attitudes and efforts made by school administrators and staff to increase competencies, explore innovations, and engage in activities that can enhance program quality. Whereas public schools and Head Start programs provide teachers with professional development opportunities and dedicated time, most childcare centers lack the financial and staffing resources to do so (Whitebook et al., 2018). Teacher perceptions of school-based professional development supports (e.g., provision of coaching) predicted implementation dose and quality of a new elementary school program (Ransford et al., 2009), suggesting that organizational learning may function similarly to support new preschool programming.

#### Workplace challenges

Conceptually, working in a well-run center characterized by predictable schedules, stable staffing, and strong collegial working relationships should increase teacher willingness and capacity to invest effort in new program implementation (Domitrovich et al., 2008). Center directors with the resources and administrative skills necessary to support the effective day-to-day management of the organization are well-positioned to provide the oversight and support needed for intervention implementation (Baker et al., 2010). However, community-based childcare center directors are often significantly under-resourced and belabored by the day-to-day challenges of recruiting and retaining high-quality teachers, attracting families, and monitoring and complying with state regulations. These kinds of workplace challenges are demoralizing and stressful for teachers and can interfere with their ability to provide consistent programming, as well as decrease their motivation to invest in new programming (Baker et al., 2010; Hunter and Bierman, 2020). Supporting this hypothesis, Baker et al. (2010) found that teacher perceptions of a supportive, collegial, and fair work climate predicted adherence, reflected in the number of Building Bridges intervention activities delivered.

## Scaling school readiness: Predicting implementation of REDI in childcare centers

Originally evaluated in Head Start centers, the REDI program was recently adapted for use in childcare centers. REDI is an evidence-based, multi-component curricular enrichment program targeting social–emotional and early literacy skills. The foundation for REDI is a social–emotional curriculum, Preschool PATHS (Domitrovich et al., 2007), which includes scripted lessons targeting social–emotional skills. REDI added a daily interactive reading program that uses books linked to the PATHS lessons designed to support oral language skill development, along with a Sound Games program to promote phonological awareness and alphabet center activities to build print awareness. A randomized controlled trial of REDI in Head Start classrooms produced positive effects on teaching quality and child outcomes in both social–emotional and language-literacy domains (Bierman et al., 2008) with sustained child benefits through ninth grade (Bierman et al., 2021).

Adaptations to REDI were made to accommodate the less-centralized structure of childcare centers and facilitate program scalability. First, given that online PD can reduce the burden of training (Powell et al., 2010; Piasta et al., 2012), the REDI training sequence for childcare teachers was reduced to two face-to-face workshop days supplemented with four online learning modules that teachers could review at their convenience. In addition, recognizing the difficulties childcare centers face in accessing professional coaches, REDI used a novel model of PD support that trained center directors to serve as coaches for their teachers. Directors attended the teacher trainings and were also provided with a one-day workshop and three online modules demonstrating the REDI coaching model (for more detail, see Hunter and Bierman, 2020). Directors held regular meetings with teachers during the implementation year to provide supportive and corrective feedback. Directors were supported by REDI Consultants who visited centers once a month to provide technical assistance and answer questions.

## The current study

The current study explored teacher characteristics and workplace factors that may have affected the implementation of REDI in childcare centers. Implementation outcomes included: (1) adherence, reflecting the number of REDI lessons and activities that were delivered, (2) quality of REDI curriculum delivery, reflecting the quality with which the lessons and activities were delivered, (3) quality of generalized teaching strategies, reflecting the overall use of REDI-prescribed teaching practices in the classroom, and (4) plans to sustain REDI implementation in the future. Based upon prior research linking teaching attributes to implementation quality (Phillips D. A. et al., 2017; Marti et al., 2018; Sutherland et al., 2018), it was hypothesized that *teacher characteristics,* especially baseline teaching skills and teacher receptivity to the intervention would predict implementation quality, including the quality of REDI curriculum implementation and the more generalized use of REDI teaching strategies in the classroom. Given that workplace factors may be especially relevant for program completion in childcare centers which are often under-resourced, it was hypothesized that *workplace factors*, including classroom resources, job satisfaction, organizational learning, and workplace challenges would predict implementation adherence, reflecting the amount of the REDI program that was delivered. We also explored the possibility that workplace factors would affect implementation quality. Finally, we explored the degree to which teacher characteristics and workplace factors might affect enthusiasm for and plans to continue REDI implementation in subsequent years.

## Materials and methods

### Design overview

During three successive years (2015–2017), licensed childcare centers serving preschool children in ten Pennsylvanian counties were sent emails describing the study. To be included, centers had to have: (1) at least one classroom that served at least five children of prekindergarten age, (2) a full-time director who could serve as a program coach, (3) an organized, regular daily schedule of activities (e.g., not a drop-in center or unstructured day care), and (4) not currently be using a formal curriculum-based social–emotional learning program. Each year enrolled childcare centers were stratified by county and size (number of classrooms) and then randomized at the center level to either the intervention or “usual practice” control condition. This study focused on the centers randomized to the intervention condition. Teachers provided information about their education and teaching experience, and classroom observations were conducted prior to intervention initiation to assess baseline teaching skills. A certified REDI trainer (the fifth author) provided intervention training to center directors and teachers in October and coordinated the intervention delivery. Classroom teachers implemented the intervention through April, with local coaching provided by their center directors. Regional REDI consultants (experienced educators trained in the REDI program and coaching process) visited centers twice monthly for the first 2 months and monthly thereafter. They met with the center director to discuss teacher progress and offer coaching support. They also observed REDI lessons and rated the quality of intervention delivery. Post-intervention classroom observations were collected in May. The guidelines for the ethical conduct of research developed by the American Psychological Association were followed throughout this study, and all procedures were approved by the University’s Institutional Review Board.

### Participants

Preschool teachers (*N* = 63) from 37 childcare centers provided data for the current study. Teachers were predominantly female (98%) and White (89%; 5% Biracial; 4% Black; 2% Latinx; < 1% Asian). They varied in age between 22 and 60 years of age (*M* = 35.6; SD = 10.7). A small sample of teachers co-taught (9.5%) and all teachers were in classrooms with at least five children who were eligible to start kindergarten in the following year. The 37 center directors were 100% female and predominantly White (90%; 7% Black; 3% multiracial). Directors’ ages ranged from 25 to 65 years (*M* = 41 years, SD = 8.73), they had between 1 and 21 years of experience as directors (*M* = 6 years, SD = 6.30) and varied in education (23% Associate degree, 26% Bachelor’s degree, 52% some graduate training or degree). Two directors were replaced during the study, one just before the intervention period began, and the other mid-intervention. The majority of centers only had one participating preschool classroom (*n* = 30; centers with two preschool classrooms *n* = 7).

### Measures

Predictors of implementation included teacher characteristics and workplace factors. Measures of implementation included adherence, quality of the REDI program delivery, quality of the generalized REDI teaching strategies, and plans for future REDI implementation.

### Teacher characteristics

Teacher characteristics that served as predictors of program implementation included education, experience, baseline teaching practices, and receptivity to intervention.

#### Experience and education

Teachers self-reported the number of years they had taught in a preschool classroom (*M* = 7.24 years, SD = 5.96, range 1–24 years). Teachers also self-reported their highest level of education on an 8-point scale (1 = less than high school, 0%; 2 = high school diploma or GED, 1.8%; 3 = Some training beyond high school but not a degree, 19.3%; 4 = one-year vocational training certificate, 5.3%; 5 = two-year Associate’s degree, 14.0%; 6 = four-year Bachelor’s degree, 33.3%; 7 = some graduate coursework, 19.3%; 8 = graduate degree, 7.0%; *M* = 5.44, SD = 1.60).

#### Baseline teaching practices

The quality of teacher–student interactions was evaluated during pre-intervention observations using the Classroom Assessment Scoring System for Pre-K (CLASS Pre-K; Pianta et al., 2008a). Trained research staff who were naïve concerning the intervention and intervention/ control group center assignment observed teachers for four 20-min periods, rating teacher-student interactions after each period on the ten items of the CLASS Pre-K. Items were rated using a 7-point scale. Three items reflected teacher efforts to promote learning and support children’s academic development (concept development, quality of feedback, and language modeling) and were averaged across the four observation periods to represent *Instructional Support* (*α* = 0.94; *M* = 2.69; SD = 0.98). Four items reflected teacher efforts to promote prosocial behaviors and social–emotional development (positive climate, negative climate, teacher sensitivity, and regard for students’ perspectives) and were averaged across the observation periods to represent *Emotional Support* (*α* = 0.79; *M* = 5.74; SD = 0.83).

Observers also rated the quality of classroom language use at baseline using the Classroom Language and Literacy Environment Observation (CLEO; Holland Coviello, 2005). CLEO observations occurred separately from the CLASS observations but were conducted by the same observers and often on the same day. They involved 20-min sessions during book reading, free play, and snack/lunch time. During each period, observers coded all teacher utterances directed toward children, identifying teacher directives/commands, questions, and other comments/statements. A total *Non-directive Talk* score was calculated by summing all questions and comments/statements across the three settings (*α* = 0.51; *M* = 22.42; SD = 6.08). In addition, after each 20-min observation, research assistants used a 5-point scale (1 = *never*; 5 = *always*) to rate the quality of teacher’s talk in areas of vocabulary use, elaboration, cognitive challenge, and decontextualized language. These scores were averaged across items and across the three settings to reflect *Richness of Talk* in the classroom (*α* = 0.91; *M* = 1.81; SD = 0.69).

Preliminary analyses showed that these different dimensions of teaching practices showed moderate to high levels of inter-correlation (*r* = 0.33–0.78; *p* < 0.05) and similar patterns of association with implementation. Hence, an overall score reflecting *Positive Teaching Practices* was calculated by standardizing and averaging scores from the *Instructional Support* and *Emotional Support* dimensions of the CLASS Pre-K and the *Non-directive Talk* and *Richness of Talk* dimensions of the CLEO (*α* = 0.76; *M* = 0.00; SD = 0.80; range = −2.21–1.85).

#### Receptivity to intervention

Center directors and REDI consultants each rated teachers’ receptivity to intervention. Ratings measured the frequency of positive teacher responses (1 = *almost never*; 5 = *almost always*) during coaching sessions with directors and consultation sessions with REDI consultants. Director (*M* = 4.72; SD = 0.32; range = 3.67–5.00) and consultant ratings (*M* = 4.43; SD = 0.58; range = 2.43–5.00) were positively skewed and were within one point of one another 87% of the time. Director and consultant scores were averaged to create an overall rating of teacher intervention receptivity (*r* = 0.25, *p* = 0.09; *M* = 4.59; SD = 0.37; range = 3.19–5.00).

### Workplace factors

Workplace factors included measures of classroom resources, teacher job satisfaction, organizational learning, and workplace challenges.

#### Classroom resources

Observers documented classroom resources using the CLEO Literacy Environment Inventory (LEI). They rated 16 items describing the number of books and writing materials in the classroom, and three items describing the degree to which literacy-related activities were displayed (e.g., “is there an area that is designated just for book reading?”; “how many varieties of paper are available for writing?”). Items were rated on a 3-point scale (0–2). We standardized and averaged the literacy environment and literacy activities scores to create an overall classroom resources variable (α = 0.76; *M* = 0.00; SD = 0.92; range = −2.95–2.06).

#### Job satisfaction

Teachers rated their overall job satisfaction using an 11-item scale developed by Gill et al. (2007). Using a 5-point scale (1 = *very dissatisfied*; 5 = *very satisfied*) teachers rated their satisfaction with their salary and benefits, workload, role, and job responsibilities (α = 0.85). Scores were averaged across the 11-items (*M* = 2.71; SD = 0.60; range = 1.00–4.00).

#### Organizational learning

Teachers completed a 7-item rating scale to describe their center’s orientation toward innovation and professional development. Items reflected staff orientation toward and support for program improvement (e.g., “In this early childhood program teachers and other professional staff… are encouraged to stretch and grow; are continually learning and seeking new ideas; respect those who take the lead in program improvement efforts; Bryk et al., 1999). Items were rated on a 5-point scale (*strongly agree* to *strongly disagree*) and summed for a total score (*M* = 3.15; SD = 0.67; *α* = 0.90; range = 2.00–4.00).

#### Workplace challenges

At the end of the intervention year qualitative interviews were conducted with teachers to discuss their experiences with the REDI intervention (see Hunter and Bierman, 2020 for a full report of these interviews). Participants were asked several questions about their workplace and colleagues. Following recommendations from Creswell and Plano Clark (2011) and Campbell et al. (2013), the first author and a graduate student undertook an iterative process for qualitative coding by clustering quotes from the interviews into thematic categories, discussing discrepancies, and reaching codebook consensus. The final codes reflected workplace challenges in the areas of staffing, scheduling, and professional development/supervision, as well as personal stress/overwork (independent coding kappa = 0.72; see Hunter and Bierman, 2020). A variable was calculated representing the proportion of workplace challenges mentioned relative to overall comments made about the workplace (*M* = 0.60; SD = 0.17; range = 0.24–0.90).

### REDI program implementation outcomes

Implementation of the REDI program included adherence (percentage of lessons and activities that were delivered) and program quality (quality of REDI teaching strategy use during REDI curriculum delivery and also generalized throughout the day). We also evaluated teacher and director plans to sustain REDI implementation in future years.

#### Program adherence

Adherence was measured using teacher reports of the REDI lessons completed during each week. Adherence scores were calculated for each component of REDI (i.e., Preschool PATHS lessons, interactive reading, Sounds Games, and alphabet center) and aggregated to reflect a teacher’s overall adherence to the program delivery plan. Adherence was calculated at the individual teacher level; adherence rates from teachers who left centers mid-year before having the opportunity to fully implement REDI were excluded from analyses. The number of lessons delivered over the course of the year were summed and divided by the total number of REDI lessons to calculate overall adherence as a percentage of the program that was delivered per teacher (*M* = 73.15%; SD = 28.13%; range = 3.86–98.00%).

#### Quality of delivery

Two aspects of REDI program delivery quality were measured: the quality with which teachers delivered the components of REDI (i.e., quality of REDI curriculum delivery) and the quality with which they used REDI teaching strategies throughout the day (i.e., quality of REDI teaching strategy use). In both areas, quality of delivery was rated by trained REDI consultants who observed teachers regularly throughout the school year. Consultants visited centers twice per month during the first 2 months of the academic year, and once per month thereafter. At each visit, consultants made efforts to watch teachers delivering the various components of REDI (e.g., Preschool PATHS, interactive reading, sound games, and alphabet center). They rated curriculum delivery quality using a 7-point scale ranging from *poor* to *exemplary* implementation. Scores were averaged across all four components to create an overall score (α = 0.86; *M* = 5.23; SD = 1.06; range = 2.00–6.86).

REDI consultants also rated teachers on the quality with which they used REDI teaching strategies in generalized ways throughout the day. Specifically, REDI consultants rated teachers on their demonstration of each of 5 teaching strategies (positive classroom management, sensitivity and responsiveness, emotion communication and support, positive limit-setting, and richness of talk) using a 5-point scale. Sample items included: “teacher encourages children to communicate how they feel, particularly when they are upset. He/she validates the children’s feelings when they are expressed” (emotion communication and support) and “the teacher is physically and mentally available to children in the setting” (sensitivity and responsiveness). An overall score for quality of REDI teaching strategy use was created by averaging scores over time across the five core REDI teaching strategies (α = 0.94; *M* = 4.22; SD = 0.54; range = 2.78–5.00).

#### Intentions for future REDI implementation

At the end of their intervention year, teachers and directors completed a 5-item scale describing their personal enthusiasm for the continued use of the REDI program in the future, and the degree to which they and their center colleagues value the program and support continued use in the future. Items were rated on a 5-point scale (from not at all to very much) and averaged to represent overall intentions for future program use (*α* = 0.86; *M* = 3.56; SD = 0.87; range = 2.00–5.00).

### Plan for analysis

As an initial step, we accounted for missing data by conducting multiple imputation (MI) with ten iterations with all variables of interest included in the model using SPSS version 26. MI is a Monte Carol technique where missing data is replaced with estimated values based on the available data and is preferable over single imputation methods of accounting for missing data (e.g., mean replacement; Graham, 2009). All analyses were conducted with imputed data.

We first conducted bivariate correlations with all variables of interest to gain a better understanding of associations between the independent and dependent variables. We then conducted a set of four multiple regressions predicting the four REDI implementation outcomes: adherence, quality of REDI curriculum delivery, quality of REDI teaching strategies used, and intentions for future REDI implementation. Predictors included teacher characteristics (teacher experience and education, positive teaching practices, and receptivity to intervention) and workplace factors (classroom resources, job satisfaction, organizational learning, and workplace challenges). Because a small number of teachers shared directors, regression analyses included robust standard errors to account for clustering (Hayes and Cai, 2007). While multilevel models were another option to account for the nested data, this approach could potentially produce biased estimates given the small sample size, number of clusters, and small intraclass correlations (i.e., < 0.001) in the current study (see Musca et al., 2011; McNeish and Stapleton, 2016). Regression analyses controlled for study cohort, county, and if the teacher had a co-teacher in the classroom with the rationale that co-teaching may have a positive impact on implementation (Shim et al., 2004). Finally, because the relatively high number of predictors in our regression models (i.e., 11) may have inflated the *R*^2^ values (see Akossou and Palm, 2013), we conducted regression models for teacher characteristics (4 variables) and workplace factors (4 variables) separately to produce *R*^2^ values specific to each of these constructs.

## Results

### Preliminary analyses

Bivariate correlations are presented in Table 1. Consider first the correlations evident among the teacher characteristics and workplace factors studied as predictors of implementation. More experienced teachers tended to be less receptive to the intervention and less satisfied with their jobs than less experienced teachers. Teachers in more well-resourced classrooms displayed more positive teaching practices at baseline than teachers in less well-resourced rooms. Job satisfaction, organizational learning, and workplace challenges were significantly inter-related, with job satisfaction higher in centers that supported organizational learning and faced fewer negative workplace challenges relative to positive workplace experiences.

**Table 1:** Correlations among all variables.

	1	2	3	4	5	6	7	8	9	10	11
Teacher Experience	–										
Teacher Education	−0.02	–									
Baseline Teaching Practices	−0.00	0.23	–								
Receptivity to Intervention	−0.35^*^	−0.02	0.10	–							
Classroom Resources	0.03	0.19	0.50^*^	−0.07	–						
Job Satisfaction	−0.26^✝^	0.01	−0.16	0.21	−0.12	–					
Organizational Learning	−0.11	−0.01	−0.13	0.29	−0.08	0.52^*^	–				
Workplace Challenges	0.04	−0.01	0.18	−0.14	−0.09	−0.47^*^	−0.39^*^	–			
Program Adherence	0.01	0.09	−0.07	−0.09	0.26	0.27	0.06	−0.56^*^	–		
Quality of Curriculum Delivery	−0.22	0.22	0.47^*^	0.50^**^	0.54^***^	0.15	0.20	−0.23	0.20	–	
Quality of Teaching Strategies	−0.25^✝^	0.16	0.30^✝^	0.61^***^	0.25^✝^	0.26	0.26	−0.23	0.02	0.86^***^	–
Intentions for Future REDI Use	−0.02	−0.15	−0.22	0.22	0.03	0.19	0.38^*^	−0.51^*^	0.32	0.31	0.31^✝^

✝ p < 0.10; * p < 0.05; ** p < 0.01; *** p < 0.00.

Next, consider correlations linking these variables with the implementation outcome measures. Intervention adherence had only one significant relationship: teachers in centers that faced fewer workplace challenges showed higher adherence delivering the REDI lessons than teachers in more organizationally challenged centers. The quality of REDI curriculum delivery was associated with baseline positive teaching practices, teacher receptivity to the intervention, and classroom resources. The quality of REDI generalized teaching strategies tended to be negatively correlated with teacher experience and positively correlated with positive teaching practices, receptivity to the intervention, and classroom resources. Finally, intentions to continue REDI implementation in the future was significantly associated with organizational learning and fewer workplace challenges.

### Regression analyses

Separate regression analyses that included the full set of teacher characteristics and workplace factors and control variables were conducted predicting each of the four implementation outcomes. Table 2 includes results from the regression analysis predicting program adherence. Teacher characteristics explained 1% of the variance and no teacher characteristics were uniquely associated with adherence. However, workplace challenges explained 42% of the variance and fewer REDI lessons were completed by teachers experiencing multiple workplace challenges compared with teachers who contended with fewer workplace challenges.

**Table 2:** Regression analyses predicting program adherence.

Variable	*B*	SE *B*	*β*	*t*	*p*
*Teacher characteristics*					
Teacher experience	−0.18	0.67	−0.07	0.27	0.79
Teacher education	−1.39	2.20	−0.05	0.63	0.53
Baseline teaching practices	−3.11	4.90	−0.08	0.64	0.53
Receptivity to intervention	−9.56	10.71	−0.11	0.89	0.37
*Workplace factors*					
Classroom resources	6.19	5.33	0.17	1.16	0.25
Job satisfaction	6.05	9.08	0.03	0.67	0.51
Organizational learning	−6.14	7.76	−0.06	0.79	0.43
**Workplace challenges**	**−57.43**	**23.05**	**−0.32**	**2.49**	**0.02**

Adjusted *R*^2^ for only teacher characteristics = 0.01; adjusted *R*^2^ for only workplace factors = 0.42.

Table 3 includes regression results from the model predicting the quality of REDI curriculum delivery. Teacher characteristics predicted 45% and workplace factors predicted 41% of the variance in quality of REDI curriculum delivery and there were multiple unique predictors. Positive baseline teaching practices and receptivity to intervention were positively associated with the quality of REDI curriculum delivery, indicating that teachers who were more skilled and receptive to consultation delivered the REDI lessons with higher levels of quality compared to less skilled teachers and teachers who were less receptive to consultation. In addition, classroom resources were positively associated with curriculum delivery quality; teachers with more classroom resources were able to implement REDI lessons with higher quality.

**Table 3:** Regression analyses predicting quality of REDI curriculum delivery.

Variable	*B*	SE *B*	*β*	*t*	*p*
*Teacher characteristics*					
Teacher experience	0.00	0.02	0.00	0.07	0.95
Teacher education	0.07	0.09	0.12	0.81	0.42
**Baseline teaching practices**	**0.29**	**0.17**	**0.36**	**1.72**	**0.02**
**Receptivity to intervention**	**1.47**	**0.38**	**0.55**	**3.81**	**< 0.001**
*Workplace factors*					
**Classroom resources**	**0.44**	**0.18**	**0.40**	**2.44**	**0.02**
Job satisfaction	0.03	0.27	0.03	0.12	0.91
Organizational learning	0.07	0.23	−0.01	0.31	0.76
Workplace challenges	−0.39	0.81	−0.09	0.48	0.63

Adjusted *R*^2^ for only teacher characteristics = 0.45; adjusted *R*^2^ for only workplace factors = 0.41.

Teacher characteristics predicted 40% and workplace factors predicted 18% of the variance in quality of generalized use of REDI teaching strategies in the classroom (Table 4). Positive baseline teaching practices and receptivity to the intervention were both significant, unique predictors of generalized REDI teaching strategy use. Teachers who were more skilled and more receptive to coaching used REDI teaching strategies with higher quality throughout the day compared to less skilled teachers and those who were less open to REDI coaching.

**Table 4:** Regression analyses predicting quality of REDI teaching strategies.

Variable	*B*	SE *B*	*β*	*t*	*p*
*Teacher characteristics*					
Teacher experience	0.01	0.01	0.10	0.77	0.44
Teacher education	0.01	0.05	0.02	0.29	0.77
**Baseline teaching practices**	**0.15**	**0.10**	**0.35**	**2.26**	**0.02**
**Receptivity to intervention**	**0.89**	**0.25**	**0.62**	**3.60**	**<0.001**
*Workplace factors*					
Classroom resources	0.12	0.10	0.20	1.20	0.24
Job satisfaction	0.17	0.14	0.21	1.19	0.24
Organizational learning	−0.04	0.13	−0.11	0.29	0.77
Workplace challenges	−0.18	0.46	−0.09	0.39	0.70

Adjusted *R*^2^ for only teacher characteristics = 0.40; adjusted *R*^2^ for only workplace factors = 0.18.

The results from the regression predicting intentions for future REDI implementation are presented in Table 5. Overall, teacher characteristics explained 13% and workplace factors explained 27% of the variance in intentions for future REDI use. The only significant unique predictor in this regression model was workplace challenges; teachers and directors in centers characterized by fewer organizational challenges felt more positively about continuing to use REDI in the future than those in centers that faced more organizational challenges.

**Table 5:** Regression analyses predicting intentions for future REDI implementation.

Variable	*B*	SE *B*	*β*	*t*	*p*
*Teacher characteristics*					
Teacher experience	0.01	0.03	0.05	0.43	0.67
Teacher education	−0.13	0.11	−0.22	1.18	0.24
Baseline teaching practices	−0.04	0.30	0.02	0.12	0.91
Receptivity to intervention	0.40	0.47	0.16	0.85	0.40
*Workplace context*					
Classroom resources	−0.01	0.25	−0.03	0.03	0.98
Job satisfaction	−0.38	0.44	−0.25	0.86	0.39
Organizational learning	0.29	0.33	0.18	0.89	0.38
**Workplace challenges**	**−2.44**	**1.04**	**−0.50**	**2.35**	**0.03**

Adjusted *R*^2^ for only teacher characteristics = 0.13; adjusted *R*^2^ for only workplace factors = 0.27.

## Discussion

Evidence-based programs that enrich preschool classroom curricula and provide teachers with professional development supports can improve classroom quality (Yoshikawa et al., 2013; Phillips D. A. et al., 2017). Such programming could be especially helpful for community-based childcare centers, which are often underserved by evidence-based supports (Larose et al., 2020) and yet are increasingly expected move the mark on readiness skills across literacy, numeracy, and social–emotional domains (Markowitz et al., 2018). In this study, we examined predictors of implementation for an evidence-based school readiness program, REDI, that targets early literacy and social–emotional skills and was adapted to support delivery in community-based childcare centers.

Results supported the first hypothesis that baseline teaching skills and teacher receptivity to the intervention would support high-quality program implementation but not adherence. Baseline teaching practices and receptivity to intervention emerged as important teacher predictors of the quality of REDI lesson delivery and REDI teaching strategy use. In contrast, none of the teacher characteristics studied were significantly associated with intervention adherence.

Study findings also supported the second hypotheses that workplace factors would support intervention adherence. Workplace factors were significantly associated with intervention adherence defined as the proportion of REDI activities completed (*R^2^* = 0.42). Interestingly, workplace factors were also significantly associated with quality of curricular delivery and intentions to implement REDI again in the future. Associations with program adherence and implementation quality are discussed further in the following sections.

### Predicting adherence to the REDI program

Curriculum adherence is one key ingredient to enriching classrooms in effective school readiness programs like REDI (Jenkins and Duncan, 2017; Nguyen et al., 2018); study findings suggest that the workplace challenges that characterize some childcare centers can undermine efforts to implement evidence-based programming. When asked to describe their workplace in an open-ended interview, childcare teachers who focused on organizational challenges at their centers such as insufficient funding, enrollment instability, staffing/turnover concerns, low-quality programming, and unsupportive or ineffective administration were likely to show reduced adherence (see Hunter and Bierman, 2020 for details about these interviews). These kinds of workplace challenges are amplified in childcare centers relative to publicly-funded programs because childcare centers often do not have consistent funding mechanisms or incentives to promote staff longevity and workforce professional development (Whitebook et al., 2018). On the other hand, teachers who reported fewer challenges and more well-resourced and effective workplaces implemented more of the REDI lessons. Teachers who faced higher levels of workplace challenges described unpredictable and stressful workdays along with less administrative and collegial support, which affected their morale and ability to prioritize delivering a new program. The fact that workplace challenges singularly predicted adherence in a model that also included individual teacher characteristics suggests that teachers were able to complete REDI lessons regardless of their background and training, a finding that has important policy implications (e.g., focusing quality standards beyond on educational attainment alone).

### Predicting quality of REDI implementation

Two facets of implementation quality were assessed by the REDI consultants: the quality with which teachers delivered the REDI curriculum components and the quality with which they utilized the REDI-prescribed teaching strategies throughout the day. Quality of REDI curriculum delivery ratings (*M* = 5.23) were higher on average than quality of generalized teaching strategy use (*M* = 4.22), likely because the guided lesson plans provided a helpful scaffold to support high-quality delivery (Phillips D. A. et al., 2017). However, the two aspects of implementation quality were highly related (*r* = 0.86). Predictors of both of these dimensions of implementation quality included two teacher characteristics—baseline levels of positive teaching skills and a teacher’s receptivity to the intervention consultation and coaching. Strong teaching skills can enable preschool teachers to more easily adopt new strategies, given their classrooms are already well-managed (Phillips D. A. et al., 2017), and learning new skills is not overwhelming. Receptivity to the intervention may have directly enhanced delivery quality by increasing teacher responsiveness to director input and feedback and by promoting efforts to adopt the recommended teaching strategies. Alternatively (or in addition), this finding could indicate other indirect influences on implementation quality such as a positive director–teacher working relationship (Baker et al., 2010), administrative support for the intervention (Langley et al., 2010; Wanless and Domitrovich, 2015), or stronger teacher “buy-in” (Locke et al., 2019). Interestingly, teacher experience was negatively correlated with receptivity to the intervention, suggesting that more experienced teachers were less responsive to the coaching process. Some past research suggests that more experienced teachers generally rate new programs as less acceptable than newer teachers (Witt et al., 1984; Ghaith and Yaghi, 1997), possibly because they feel settled and comfortable with their established practices.

In addition to these teacher characteristics, workplace factors, specifically classroom resources, also predicted the quality of REDI lesson delivery. Presumably, better-resourced classrooms provide teachers with access to higher quality learning materials which they can utilize to structure effective learning experiences.

### Intentions for future REDI implementation

Intentions to use REDI in the future is not a direct measure of implementation quality but represents an important implementation outcome indicating perceptions of a REDI’s sustainability. Preschool administrators and teachers make a significant investment when they initiate new evidence-based programming into classrooms. Prior research suggests that initial implementation experiences play an important role in determining whether preschools leverage this initial investment with sustained program use (Bierman et al., 2013). In this study, workplace factors emerged as the critical predictors of intentions for future REDI program implementation. Organizational learning and workplace challenges each predicted future intentions to use REDI, with workplace challenges contributing unique variance in the regression model. In this study, childcare center workplace challenges emerged as a barrier to implementation adherence, quality of curriculum delivery and teaching strategy use, as well as intentions for future REDI use. These findings suggest that improving childcare program quality will require addressing the day-to-day management struggles faced by under-resourced childcare centers (Whitebook et al., 2018) which represent critical barriers to the effective implementation of enriched curricula and uptake of professional development training.

### Implications for evidence-based interventions in childcare centers

Early childhood stakeholders including program administrators, researchers, and policy-makers are concerned with bringing effective school readiness interventions to scale, which necessarily requires an understanding of their implementation determinants. As school-based SEL program implementation ramps up in a variety of learning contexts (Bryant et al., 2021), questions remain about how school readiness programs like REDI are being delivered in practice. To that end, this study has several implications for scaling evidence-based programs in childcare centers serving preschool children. First, workplace factors require more attention in research and more emphasis in policy-related efforts to improve childcare. In the previous study of REDI implemented in the Head Start context, workplace climate perceptions were not related to implementation quality (Domitrovich et al., 2019). In contrast to Head Start, the characteristics of childcare center workplaces may be especially variable given the range of organizational infrastructures that support them, including private owners, nonprofit cooperatives, and faith-based organizations (Ackerman and Sansanelli, 2010; Bassok et al., 2016a). Workplace variation also exists as a function of the financial stressors and unstable teacher and student base in many childcare centers, as well as the diverse cultural expectations in different communities served (Ackerman and Sansanelli, 2010). Attention paid to the workplace environment in centers may help assess readiness for implementation (e.g., Wanless and Domitrovich, 2015), a factor that itself may need to be a target of intervention in some cases. Policy efforts aimed at expanding childcare center opportunities for preschool children (e.g., Child Care and Development Block Grants; Guarino, 2021) should also note that over-burdened and under-resourced centers may require more structural assistance (e.g., incentives to retain staff; expanded professional development; classroom resources) to assist teachers with implementing high-quality preschool programming.

In addition, this study has implications for improving and scaling professional development efforts for childcare center teachers. The professional development model evaluated in the original Head Start REDI study (Bierman et al., 2008) was adapted in this study to provide a more cost-effective and scalable model for childcare centers. Innovations included the use of online learning modules to reduce time spent in in-person workshops which are challenging for childcare centers to finance, and the use of center directors as coaches for their teachers. To help teachers deliver the REDI curriculum with high levels of quality and use the generalized teaching strategies throughout the day, directors met with them routinely for goal-setting, problem-solving, and mentoring and also observed their implementation of REDI lessons to provide feedback. Teachers’ receptivity to this consultative process emerged as an important predictor of implementation, over and above other teacher characteristics such as education and experience. This result supports the expectation that engagement with and receptiveness to coaching is highly relevant for implementation outcomes, which can improve classroom quality. Indeed, a prior study of MyTeachingPartner (Pianta et al., 2008b) showed that teacher responsiveness to intervention (coach ratings of engagement) mediated the association between perceived intervention quality (teacher ratings) and changes in teacher-child interactions (LoCasale-Crouch et al., 2016). In the current study, center directors reported that although it was difficult to find time for coaching, they considered it a valuable process (Hunter and Bierman, 2020). As such, an emphasis on building infrastructure to support coach-mentors (directors or other qualified staff) in childcare centers seems warranted and would be improved by boosting teachers’ readiness to engage with such coaching.

Finally, past research examining teacher professional experience with successful program implementation has yielded mixed results. In reviewing the literature, we found that teachers with advanced education implemented some preschool interventions with higher quality than teachers with less formal education (Williford et al., 2015; Sutherland et al., 2018) whereas teacher education levels showed no association with implementation quality in a number of other studies (Baker et al., 2010; Wenz-Gross and Upshur, 2012; Phillips B. M. et al., 2017). Associations between teacher education levels and implementation adherence and quality were also non-significant in the present study. At the same time, current policy efforts target regulating structural features of preschool programs such as teacher education levels in an effort to equalize early childhood care across sectors (Child Trends, 2019), although empirical relations among such structural features to child outcomes are weak (Early et al., 2007; Farran and Hofer, 2011; Perlman et al., 2017). Given that evidence-based programming and professional development can strengthen process quality (i.e., teacher-child interactions), which is more consistently related to lasting child outcomes (Phillips D. A. et al., 2017), emphasizing program curricular enhancements and PD may be a more effective and cost-efficient target for policy efforts than structural regulatory control. However, with the relatively sparse research base and mixed results, more research is needed to determine which facets of program implementation are assisted by teacher education levels or other structural characteristics of childcare centers, especially amid calls to standardize the training, educational requirements, and wages of pre-K through third grade teachers (Whitebook et al., 2018).

## Limitations and conclusions

This study represents an initial evaluation of individual and workplace factors related to teachers’ implementation of an evidence-based curricular enhancement intervention for preschool children in childcare centers. Additional research examining predictors of implementation quality for these kinds of program enhancements could inform the challenge of scaling up high-quality early education and reducing disparities in quality often experienced by children living in under-served communities and settings (Phillips B. M. et al., 2017; Nguyen et al., 2018). Given that access to early childhood programs has increased dramatically in recent years but quality remains inconsistent and often low (Friedman-Krauss et al., 2014; Barnett et al., 2017; Child Trends, 2019), research targeting processes that can improve the scale-up of high-quality programming across the many settings where young children are served is vital.

This study adds to limited previous work examining preschool interventions in childcare centers (Larose et al., 2020) and extends it by measuring both qualitative and quantitative aspects of the workplace in addition to multiple measures of implementation. However, several limitations are noted. First, the childcare centers in this sample were all located in Pennsylvania, and although they represented diverse geographic and socio-economic areas in the state, the generalizability of the findings to centers in states with different structures supporting and regulating early care is unknown. Second, this study followed recommendations from conceptual models to measure multiple factors at the individual (teacher) implementer level as well as the contextual organizational (workplace) level to understand implementation (Domitrovich et al., 2008). However, it is possible that other latent characteristics of either the teachers, directors, or interactions in the workplace not measured in this study could explain further variance in the outcomes assessed. Further, this study did not include director buy-in as a potential influence on either the workplace climate or implementation (although other studies have considered it a workplace factor; Domitrovich et al., 2009). Future research validating this work, and potentially understanding prediction by individual aspects of the workplace (e.g., administrative leadership; Domitrovich et al., 2019) or evaluating the mechanisms through which individual and workplace factors impact child outcomes, is necessary.

Additional studies might also address some of the measurement limitations of this study. These included the narrow focus on the literacy materials in the classrooms to represent classroom resources and the fact that REDI consultants rated teacher receptivity to intervention and contributed to ratings of delivery quality, thereby possibly increasing the associations of those two variables. Finally, caution should be taken with the *R*^2^ values from the regression models. While we attempted to limit the risk of overinflating the *R*^2^ by calculating adjusted *R*^2^ for teacher characteristics and workplace factors separately, our *R*^2^ values were higher than what has been reported in a limited number of implementation studies (e.g., Ransford et al., 2009 reported *R*^2^ between 0.10 and 0.16 for implementation quality). It is possible that our sample size over-inflated the *R*^2^ (Karch, 2020) and more research is needed to better understand the amount of variance explained in implementation by teacher characteristics and workplace factors.

Although they are often overlooked in large-scale research and policy efforts, the community-based childcare professionals in this study showed positive interest and enthusiasm for the REDI program and PD model, and many were especially interested in continuing to implement the social–emotional learning aspects of REDI (Hunter and Bierman, 2020). Teachers also achieved high levels of implementation adherence and quality despite structural and workplace challenges. As such, the current study demonstrates both the viability and importance of attending to the unique characteristics of childcare centers in designing school readiness interventions and implementation supports that bolster program competence and child skills. Considerations for evidence-based policy and practice recommendations that assist childcare center professionals in making sustainable improvements like those studied here remain a priority.

## Data availability statement

The datasets presented in this article are not readily available because the authors are bound by ethical obligations that could be violated upon sharing of the original dataset. They will make the data supporting the conclusions of this article available to qualified researchers upon request, with restrictions as needed to preserve the confidentiality of participating teachers, directors, and centers. Requests to access the datasets should be directed to kb2@psu.edu.

## Ethics statement

The studies involving human participants were reviewed and approved by The Human Research Protection Program (HRPP) at Penn State. The participants provided their written informed consent to participate in this study.

## Author contributions

All authors listed have made a substantial, direct, and intellectual contribution to the work and approved it for publication.

## Funding

This work was supported by the National Institute of Child Health and Human Development under Grant number HD079410; the Institute of Education Sciences under Grant number R305B090007; and a dissertation grant from the Society for the Study of School Psychology.

## Conflict of interest

The authors declare that the research was conducted in the absence of any commercial or financial relationships that could be construed as a potential conflict of interest.

## Author disclaimer

The views expressed in this article are ours and do not necessarily represent the granting agencies.

## Publisher’s note

All claims expressed in this article are solely those of the authors and do not necessarily represent those of their affiliated organizations, or those of the publisher, the editors and the reviewers. Any product that may be evaluated in this article, or claim that may be made by its manufacturer, is not guaranteed or endorsed by the publisher.

## Supplementary material

The Supplementary material for this article can be found online at: https://www.frontiersin.org/articles/10.3389/fpsyg.2022.1023505/full#supplementary-material

## Footnotes

^1^ See Supplementary material for a table listing a description of each intervention reviewed, the predictors of implementation included in the study, and their relation to implementation.
