# Supplementary material for: Predicting school readiness program implementation in community-based childcare centers
Source: Front Psychol. 2022 Dec 15;13:1023505. doi: 10.3389/fpsyg.2022.1023505 (PMC9798309; doi:10.3389/fpsyg.2022.1023505)
Supplement: Supplementary file 1 [file Data_Sheet_1.PDF]

**LEVEL 2** – Multiple Aspects		Description	Examples
**1. Thoughts, Reflections, Memories**		**Situation-specific or more general thoughts, reflections, associations, speculations, and upcoming memories during the trial, possibly related to the content of the other categories**	"I started thinking about the task and also about my own reactions" (P 1)<br>"...and wonder if he perceives something like that in my gaze as well" (P 19)<br>"Incompleteness characterizes the encounter" (P 23)
**2. Feelings**		**Whole spectrum of one's own positive, negative, or absent/neutral feelings (except bodily induced states), and temporal experience**	
	2.1 Affective Feelings	Basic affective feelings, moods, and states; social emotions and emotional attitudes toward the other person; change of feelings	"...I also feel a little depressed in the process" (P 2)<br>"...and felt very close to her" (P 18)
	2.2 Metacognitive Feelings	Execution of the task, i.e., related cognitive activities, succeeds easily, difficulty, or fails, possibly accompanied by corresponding feelings (e.g., comfort, effort, frustration)	"At the beginning, I found it very difficult to concentrate" (P 5)<br>"Mental: overall tense process" (P 24)<br>"The experiment went much easier and I was able to concentrate better on maintaining eye contact without wandering off" (P 27)
	2.3 Sense of Time	Time seems to be stretched, or to pass quickly, e.g., in comparison of the experimental conditions	"...In the end, the time was up quickly and could have gone longer" (P 4)<br>"It felt like an eternity" (P 26)
**3. Body**		**One's own bodily sensations and reactions**	
	3.1 Sensation	Felt energy level, arousal/tension/relaxation indicated by increased heartbeat or breath; sensation of posture, etc.	"Breathing was difficult, and I felt the heat under the mask" (P 15)<br>"...initially tense and in the course very relaxing, the exercise has a very decelerating effect and does lasting good" (P 24)
	3.2 Reaction	Externally observable: Blinking, watering eyes, yawning, etc. Smiling/laughing is included if it occurs unintentionally as a (automatic) impulse and without explicit interaction context	"...had to laugh frequently ... My posture also changed: first it was rigid and somewhat tense; later it became looser and more relaxed..." (P 5)<br>"I also noticed that I hardly had to blink at all" (P 25)
**4. Observation**		**Physical appearance of the other person and attention regulation**	
	4.1 Content / Quality	Physical appearance of the interaction partner, e.g., eye color, posture, face and facial movements/expressions, emotions, becoming aware of the other; *perceptual content* appears clear/blurry/altered	"Over and over again I looked at the whole face" (P 4)<br>"Over time, the counterpart became more and more blurred" (P 6)<br>"You seem a little sad to me" (P 14)
	4.2 Attention Regulation	Direction and scope of focus; changing between focused/defosued attention; clear/blurry/altered *view* (without specific content); concentration/distraction; distanced/immersed observation	"...as both participants were distracted by the unfamiliar situation" (P 6)<br>"...this focus was primarily on the right eye of my counterpart from my perspective" (P 11)<br>"The view of the other person became more global" (P 13)
**5. Intentions**		**One's own urges, wishes, and intentions, e.g., trying to concentrate, to escape, etc., referring to individual interactions or to the entire trial; not (yet) initiated or completed forms of action, or retrospective explanation of the purpose of an action; often related to Categories 5.2 and 6.1**	"...I tried to focus on what I could see in my counterpart" (P 1)<br>"... in order to make me aware of how the person is doing" (P 17)<br>"Wanted to impulsively rip off her mask at the beginning of the silence" (P20)
**6. Behavior and Interaction**		**All forms of one's own and the partner's overt behavior during the dyadic interaction**	
	6.1 Eye Gaze	Mutual gazing, sending and receiving cues; dynamics and phenomenal quality of view	"...my eyes [were] just drawn to my partner's eyes like a magnet" (P 18)<br>"...because I have the feeling to communicate through the eyes" (P 19)
	6.2 Mirrored Behavior	Contagious smiling/laughing, yawning, tension/relaxation, mirrored posture, breath, emotions	"I have to start laughing because the other person is laughing" (P 2)<br>"...mirroring the other person, such as head posture or even the rhythm of breathing..." (VP 5)
	6.3 Other Behavior	One's own and the other's behavior beyond 6.1 and 6.2 but also in dyadic or communicative context; one's own prevented behavior (e.g., impulse control)	"...the height of the mask, which is corrected once by me" (P 3)<br>"I had to suppress my laughter all the time" (P 10)<br>"I could see the smile, the smirk and the attempt to remain serious ..." (VP 16)

Table 1. Second Coding Level
