# Supplementary material for: Predicting school readiness program implementation in community-based childcare centers
Source: Front Psychol. 2022 Dec 15;13:1023505. doi: 10.3389/fpsyg.2022.1023505 (PMC9798309; doi:10.3389/fpsyg.2022.1023505)
Supplement: Supplementary file 2 [file Data_Sheet_2.PDF]

**LEVEL 3** – Mental Micro Activities		Description	Examples
**1. Proactive / focused / self-assertive (A) – PF-A**		**Focused attention; investigative observation; proactive, extroverted, and initiative attitude in the interaction; person A is aware of herself and her intentions regarding B and thus may express herself self-assertively.**	"... First, a consideration of face, shape of nose, mouth, eyebrows, eyes, the face as a whole (thinking, observing, being with me and looking at her" (P 23)
**2. Proactive / focused / self-assertive (B) – PF-B**		**Analogous to Cat. 1 with roles reversed; from the perspective of person A, a proactively focused attitude emanating from person B is perceived; one feels looked at, fixed, or challenged; the intention directed at one can be experienced as the self-assertion of person B; uncomfort feelings or escape impulses can arise**	"... She was switching back and forth between my two eyes the whole time" (P 10)<br><br>„The long eye contact feels like a 'nakedness', as if you are giving the other person a glimpse of your inner self" (P 19)
	2.1 PF-B Protection	<u>Only mask condition</u>: Person A perceives her mask as a protection from PF-B activities or as a means of hiding her facial expressions from the other person's gaze; this can be accompanied by positive, especially relieved feelings.	„With mask I felt more comfortable and confident in the encounter, eye contact was immediately easier" (P 13)
**3. Receptive / opening / devotional (A) – RO-A**		**Person A opens up to, identifies and accepts what is coming from person B as described in Cat. 2; an inner space is given for what emanates from person B and is perceived receptively; person A surrenders to B's presence without asserting their own impulses at this moment.**	"It was as if I opened myself for a moment and made myself empty, so that the impression of the other person in me could get space" (P 13)
	3.1 RO-A Inhibition	<u>Only mask condition</u>: Person A perceives the mask of person B as obstacle for her own RO-A activity and experiences a reduced empathy. Typical expressions include the 'depth' or 'soul' of the other person, which cannot be adequately grasped.	„The experience of being able to look through the eyes into the depths of the partner was lost" (P 22)
**4. Receptive / opening / devotional (B) – RO-B**		**Analogous to Cat. 3 with roles reversed; person A experiences person B opening to and receptively receiving what emanates from herself (A); person A feels perceived, accepted, or secure; person B is perceived as being with person A and suspending her own intentions.**	"The feeling of being noticed" (P 29)<br><br>„She also noticed that in my eyes and had also gotten 'tunnel vision'" (P 32)
**5. Connection / Resonance**			
	5.1 Positive	Felt connection or resonance between the participants; binding exchange, which is less static, but rather exhibits a subtle and bidirectional dynamic; more general or symmetric than categories 1 to 4; this can be accompanied by feelings of agreement, trust, closeness, or intimacy.	„In the end, I had the impression that we were on the same wavelength" (P 20)
	5.2 Negative	The opposite of category 5.1; typical expressions are negations of connection, diminished contact, interaction difficulties, distance, isolation, and feelings of coldness or antipathy.	„...however, I felt that moment as if there was a distance between us" (P 5)

Table 2. Third Coding Level. Coding categories with subcategories, descriptions, and exemplary excerpts from the data.
