# Supplementary material for: Predicting school readiness program implementation in community-based childcare centers
Source: Front Psychol. 2022 Dec 15;13:1023505. doi: 10.3389/fpsyg.2022.1023505 (PMC9798309; doi:10.3389/fpsyg.2022.1023505)
Supplement: Supplementary file 3 [file Data_Sheet_3.PDF]

	With explicit Body Ref. (BRI = 1)	Gaze (BRI = 0.5)	Without explicit Body Ref. (BRI = 0)
**PF-A**	I start to look more closely at the person's face covering. (02/with)	... so that I had the eyes in focus as if in tunnel vision (05/without)	I wanted to be closer to you again and I formulated my feelings and sensations that came up at that moment to you. (28/with)
**RO-A**	... look into her eyes, be open to her (be with her) (23/without)	My partner returned my gaze, but she felt very uncomfortable under observation. (13/without)	I open up to the person and feel strongly involved and responsible. (17/without)
**PF-B**	She switched back and forth between my two eyes the whole time. (10/without)	I also wanted to avert my gaze for a second, because it had become very exhausting to hold the gaze towards the end. (26/without)	I feel naked, exposed (08/without)
**RO-B**	... I had the feeling she opens her eyes to me and with that also the inside, so that I can look into her soul and she has nothing to hide from me. (10/without)	She also noticed that in my eyes, she also got a "tunnel vision" and she noticed that my thoughts are completely with me ... (32/with)	... the feeling of being perceived (29/without)
**RO-A Inhib.**	The experience of being able to look through the eyes into the depths of the partner was lost. (22/with)	–	I do not experience you. (14/with)
**PF-B Prot.**	The mask was like a "protective shield" (27/with)	With mask I felt more comfortable and confident in the encounter, eye contact was immediately easier. (13/with)	I didn't feel watched and much more relaxed. (10/with)
**Conn./Res (+)**	We both realized that this task is very personal right now and the eye contact is very profound. (32/without)	The look represents a connection, almost a dependence. (17/without)	I felt a connection between us. (18/with)
**Conn./Res. (-)**	The emotional distance was greater than in the first trial, despite the strong eye contact. (07/with)	–	Didn't feel any connection between us. (20/with)

Table 4. Level 3: Varying dependence on body reference (exemplary codings). Information about Participant and condition is indicated in brackets.
