# Supplementary material for: Predicting school readiness program implementation in community-based childcare centers
Source: Front Psychol. 2022 Dec 15;13:1023505. doi: 10.3389/fpsyg.2022.1023505 (PMC9798309; doi:10.3389/fpsyg.2022.1023505)
Supplement: Supplementary file 4 [file Data_Sheet_4.PDF]

	PF-A	RO-A	PF-B	RO-B	RO-A Inh.	PF-B Prot.	Conn./Res. (+)	Conn./Res. (–)	*Mean*
**No Mask**	0.800	0.264	0.462	0.500	–	–	0.235	0.286	**0.422**
**Mask**	0.786	0.188	0.500	0.667	0.500	0.625	0.400	0.130	**0.451**

Table 5. Level 3: Body Reference Index across categories and conditions.
