# Supplementary material for: Predicting school readiness program implementation in community-based childcare centers
Source: Front Psychol. 2022 Dec 15;13:1023505. doi: 10.3389/fpsyg.2022.1023505 (PMC9798309; doi:10.3389/fpsyg.2022.1023505)
Supplement: Supplementary file 5 [file Table_1.doc]

Supplementary Material

Summary of Associations Found in Preschool Interventions Predicting Implementation Adherence or Quality

| **Intervention** | **Intervention Components** | **Predictors of Implementation Adherence and Quality** | | | | | | |
| --- | --- | --- | --- | --- | --- | --- | --- | --- |
|  |  | Teacher Characteristics | | | Workplace Functioning | | | |
|  |  | Education/  Experience | Skills | Reception | Job  Satisfaction / Burnout | Class  Resources | Org. Learning | Work Climate |
| Banking Time (Williford et al., 2015) | Brief individual teacher-child sessions focused on improving disruptive behavior; ongoing teacher consultation | AD |  |  |  |  |  |  |
| BEST in CLASS (Sutherland et al., 2018) | Teacher-delivered instructional practices used with children having behavior challenges (Tier 2 intervention). | QU | QU |  |  |  |  |  |
| BLOOM Language Curriculum (Phillips et al., 2017) | Multi-component, teacher-delivered vocabulary and language curriculum supplement. | NR | QU | AD |  |  |  |  |
| Building Bridges (Baker et al., 2010) | Brief, teacher-delivered weekly activities in social-emotional and academic domains; ongoing teacher consultation. | NR |  | AD | AD |  |  | AD |
| Getting Ready for School  (Marti et al., 2018) | Literacy, math, and self-regulation curriculum with multiple components supplement delivered in school (by teacher) and home (by parent). |  | QU |  |  |  |  |  |
| Head Start REDI (Domitrovich et al., 2009) | Multi-component literacy and social-emotional skill curriculum supplement; ongoing teacher consultation. | NR |  | QU | QU |  |  | NR |
| Language skills program: *Texas School Ready!* (Zucker et al., 2013) | Classwide book reading activities (Tier 1) with small-group targeted activities (Tier 2) |  |  | QU |  |  |  |  |
| My Teaching Partner (LoCasale-Crouch et al., 2016) | Coached teaching strategies to improve teacher-child relationship |  |  | QU |  |  |  |  |
| PATHS (Ransford et al., 2009) | Teacher-delivered social-emotional skill lessons and activities |  |  |  | AD |  | AD  QU |  |
| Second Step (Wenz-Gross and Upshur, 2012) | Teacher-delivered social-emotional skill lessons and activities | NR | AD  QU |  |  | AD |  |  |

*Note.* Cells are filled in to show significant associations with AD = adherence or QU = quality. NR= No relationship found. Blank boxes indicate that the variable was not included in the study.
